# Safety of JN.1-Updated mRNA COVID-19 Vaccines

**DOI:** 10.1001/jamanetworkopen.2025.23557

**Published:** 2025-07-28

**Authors:** Niklas Worm Andersson, Emilia Myrup Thiesson, Anders Hviid

**Affiliations:** 1 Department of Epidemiology Research, Statens Serum Institut, Copenhagen S, Denmark

## Abstract

This cohort study investigates the association between booster vaccinations containing the JN.1 lineage and 29 serious adverse events in Denmark.

## Introduction

Updated mRNA COVID-19 vaccines targeting the SARS-CoV-2 Omicron JN.1 lineage were recently authorized in the US and Europe^1,2^ and made available as the 2024-2025 season booster dose in many countries. Data to support their safety are scarce. This nationwide cohort study investigated the association between vaccination with JN.1-containing vaccines and the risk of 29 serious adverse events.

## Methods

The study cohort was established through linkage of individual-level data from nationwide health care registers. Danish law exempts register-based research in Denmark from informed consent and ethics committee approval. This study is reported following the STROBE reporting guideline.

All adults in Denmark recommended to receive the 2024-2025 JN.1-containing booster vaccine (ie, those aged ≥65 years or individuals in high-risk groups) who had previously received 3 or more COVID-19 vaccine doses were included. The study period ran from May 1, 2024, to March 31, 2025. Vaccination status during follow-up was classified as a time-varying variable (eTable in Supplement 1).

We analyzed 29 adverse outcomes adapted from prioritized lists of adverse events of special interest to COVID-19 vaccines. Each adverse event was analyzed separately. Adverse events were identified as the first hospital contact with a recorded outcome diagnosis, with the date of diagnosis considered the event date (eTable in Supplement 1).^3,4,5,6^ During the study period, individuals were followed up until first outcome event while censoring upon emigration, death, or the end of the study period. Outcome rates during the first 28 days after JN.1-containing vaccine administration (ie, the risk period) were compared with outcome rates during the remaining period (ie, the reference period, with follow-up from the study start or ≥43 days after any prior dose or the JN.1-containing vaccine dose) as previously described (eFigure in Supplement 1).^5,6^ If uncensored, individuals could contribute person-time to 28-day risk and reference periods. We compared risk and reference period outcome rates using Poisson regression to estimate incidence rate ratios adjusted for sex, age, region, vaccination priority group, calendar time, and number of comorbidities. Statistical tests were 2-sided and conducted in R statistical software version 4.1.1 (R Project for Statistical Computing). Associations were considered statistically significant if the 95% CI did not overlap 1.

## Results

The cohort totaled 1 585 883 individuals (mean [SD] age, 66.8 [14.5] years; 862 585 female [54.4%]), of whom 1 012 400 individuals (mean [SD] age, 73.5 [10.3] years) received updated mRNA COVID-19 vaccines containing the JN.1 lineage vaccine during follow-up (Table). No statistically significant increases in the rate of hospital contacts for any of 29 adverse events were observed during the 28-day risk period after receipt of a JN.1-containing mRNA vaccine compared with reference period rates (Figure). For example, the incidence rate ratio was 0.84 (95% CI, 0.76-0.94) for ischemic cardiac events, 0.92 (95% CI, 0.76-1.13) for intracranial bleeding, and 1.12 (95% CI, 0.41-3.10) for myocarditis.

**Table.** Study Cohort Characteristics

Characteristic^a^	Individuals, No. (%)		
	Entire study cohort (N = 1 585 883)	Vaccinated with JN.1-containing vaccine (n = 1 012 400)	Not vaccinated with JN.1-containing vaccine at study end (n = 573 483)
Age, mean (SD), y	66.8 (14.5)	73.5 (10.3)	60.6 (17.1)
Sex			
Female	862 585 (54.4)	547 550 (54.1)	315 035 (54.9)
Male	723 298 (45.6)	464 850 (45.9)	258 447 (45.1)
Region of residency			
Capital Region of Denmark	437 167 (27.6)	274 017 (27.1)	163 150 (28.4)
Central Denmark Region	358 459 (22.6)	233 305 (23.0)	125 154 (21.8)
Northern Denmark Region	173 947 (11.0)	110 645 (10.9)	63 302 (11.0)
Region Zealand	255 036 (16.1)	166 728 (16.5)	88 308 (15.4)
Region of Southern Denmark	361 232 (22.8)	227 693 (22.5)	133 539 (23.3)
Region not specified	42 (<0.1)	12 (<0.1)	30 (<0.1)
Vaccination priority groups			
Age priority	1 145 608 (72.2)	877 529 (86.7)	268 079 (46.7)
High-risk priority	440 275 (27.8)	134 871 (13.3)	305 404 (53.3)
Comorbidities			
Asthma	54 596 (3.4)	31 099 (3.1)	23 497 (4.1)
Chronic respiratory disorder	64 802 (4.1)	46 563 (4.6)	18 239 (3.2)
Chronic cardiac disorder	298 513 (18.8)	205 581 (20.3)	92 932 (16.2)
Kidney disorder	26 021 (1.6)	16 877 (1.7)	9144 (1.6)
Diabetes	111 169 (7.0)	64 944 (6.4)	46 225 (8.1)
Autoimmune disorder	158 145 (10.0)	85 931 (8.5)	72 214 (12.6)
Epilepsy	15 493 (1.0)	9957 (1.0)	5536 (1.0)
Malignant neoplasm	157 967 (10.0)	100 085 (9.9)	57 882 (10.1)
Psychiatric disorder	113 395 (7.2)	61 245 (6.0)	52 150 (9.1)

^a^ The start of the study period was May 1, 2024, while the rollout of the JN.1-containing booster vaccine was initiated at approximately the start of October 2024, and the study period ended on March 31, 2025.

**Figure.**
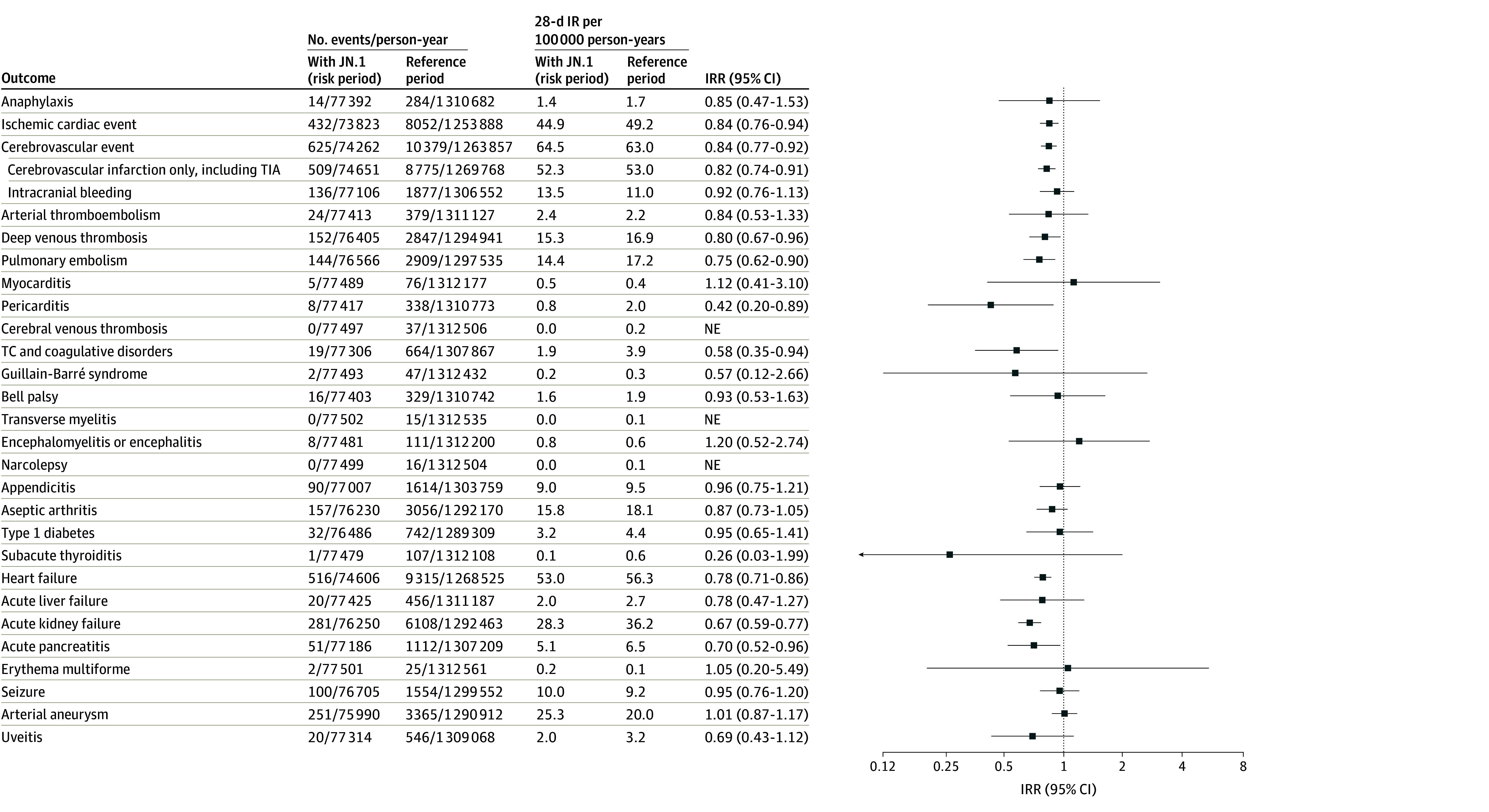
Risk of Adverse Events After Booster Vaccination With JN.1-Containing Vaccines The figure shows adjusted incidence rate ratios (IRRs) for 29 adverse events of special interest to COVID-19 vaccines; rates observed during the 28-day risk period after booster vaccination with updated mRNA COVID-19 vaccines containing the SARS-CoV-2 Omicron JN.1 lineage (KP.2 strain) are compared with rates during the reference period. Each outcome was studied separately, resulting in potential differences in denominators due to varying exclusions. IR indicates crude incidence rate; NE, not estimable; TC, thrombocytopenia; TIA, transient ischemic attack.

## Discussion

In this nationwide cohort study, no increased risk of 29 adverse events was observed after vaccination with the updated COVID-19 mRNA vaccine containing the SARS-CoV-2 Omicron JN.1 lineage in approximately 1 million adults. Limitations include that residual confounding and health care use bias cannot be excluded even with the use of within-individual comparisons. Additionally, although we analyzed a nationwide cohort, some outcomes, such as erythema multiforme, occurred very rarely during follow-up, with consequently lower statistical precision, and some outcomes, such as transverse myelitis, could not be statistically compared. Notably, the upper bound of the CI for 19 of 29 adverse events examined was inconsistent with moderate to large increases in relative risks of more than 1.5.

## Notes

Supplement 1. **eTable.** Eligibility criteria, outcome, and covariate definitions **eFigure.** Schematic figure of the study design
eReferences.

Supplement 2.
Data Sharing Statement
