# Supplementary material for: Safety of JN.1-Updated mRNA COVID-19 Vaccines
Source: JAMA Netw Open. 2025 Jul 28;8(7):e2523557. doi: 10.1001/jamanetworkopen.2025.23557 (PMC12305385; doi:10.1001/jamanetworkopen.2025.23557)
Supplement: Supplement 1. — eTable. Eligibility criteria, outcome, and covariate definitions eFigure. Schematic figure of the study design eReferences. [file jamanetwopen-e2523557-s001.pdf]

Supplemental Online Content

Andersson NW, Thiesson EM, Hviid A. Safety of JN.1-updated mRNA COVID-19 vaccines. *JAMA Netw Open*. 2025;8(7):e2523557. doi:10.1001/jamanetworkopen.2025.23557

eTable. Eligibility criteria, outcome, and covariate definitions

eFigure. Schematic figure of the study design

eReferences.

This supplemental material has been provided by the authors to give readers additional information about their work.

© 2025 Andersson NW et al. *JAMA Network Open*.

eTable 1. Eligibility criteria, outcome, and covariate definitions

Variable	Details
**Eligibility criteria**	
Aged ≥18 years (adult)	*The Civil Registration System.*[1] The register provides the mandatory unique personal identifier for all permanent residents of Denmark, allowing the cross-linkage of all Danish healthcare services and civil registration systems. The register also holds demographic information such as birthdate, sex, continuously updated information and dates on historical addresses, immigration and emigration status, and death. Age was defined by year of study entry minus birthyear. As such, to be included in our cohort, an individual had to be born in 2006 or earlier (i.e., turning 18 years or older in 2024; age was defined by year of study minus birth year.
Prioritized for 2024-2025 season Covid-19 vaccine vaccination (aged ≥65 years or within high-risk group)	*The Danish Vaccination Register.*[2] Defined according to current recommendations for populations targeted for this season's Covid-19 vaccine by the Danish health authorities: those ≥65 years of age belonging to a high-risk group of severe Covid-19 (governmentally prioritized).
Vaccinated with a primary course and one booster Covid-19 vaccine (i.e., three prior Covid-19 vaccine doses)	*The Danish Vaccination Register.*[2] Defined as registered three previous Covid-19 vaccines received with the BNT162b2 and/or mRNA-1273 as well as and/or AZD1222 vaccine (the latter as part of the primary vaccination course only). Other vaccine types were rare within our source population and were treated as censoring events. During the national rollout of the primary vaccination course, vaccination with the AZD1222 vaccine was halted in Denmark in early March 2021.
**Outcomes**	The National Patient Register.[3] Both primary and secondary diagnoses, recorded during any type of first-time hospital contact using ICD-10 codes.[a]
Anaphylaxis	ICD-10: T782, T783, T805, T886
Ischemic cardiac event	ICD-10: I20-I251
Cerebrovascular event	ICD-10: I60-66, G450-G453
Cerebrovascular infarction (incl. TIA)	ICD-10: I63, I64, G450-453
Intracranial bleeding	ICD-10: I60-I62
Arterial thromboembolism	ICD-10: I74
Deep venous thrombosis	ICD-10: I80-82 (not I800, I808C, or I821)
Pulmonary embolism	ICD-10: I26
Myocarditis	ICD-10: I401, I408, I409, I418, I514
Pericarditis	ICD-10: I300, I308, I309, I328
Cerebral venous thrombosis	ICD-10: I636, I676
Thrombocytopenia or coagulative disorders	ICD-10: D65, D683, D686, D688-689, D690, D693-D699 (not D697 or D698A)
Guillain-Barré syndrome	ICD-10: G610
Bell's palsy	ICD-10: G510
Transverse myelitis	ICD-10: G373
Encephalomyelitis or encephalitis	ICD-10: G040, G040A, G048, G049, G058, G361
Narcolepsy	ICD-10: G474
Appendicitis	ICD-10: K35-K37
Aseptic arthritis	ICD-10: M10, M119, M130, M131, M139
Type 1 diabetes mellitus	ICD-10: E10
Subacute thyroiditis	ICD-10: E061
Heart failure	ICD-10: I110, I420, I426-I429, I50, J81,

© 2025 Andersson NW et al. *JAMA Network Open*.

Acute liver failure	ICD-10: K71, K72
Acute kidney failure	ICD-10: D593, I12, I13, N00-N02, N04-N05, N08, N10, N141, N142, N144, N17, N19, R34
Acute pancreatitis	ICD-10: K850, K853, K858, K859
Erythema multiforme	ICD-10: L51
Seizure	ICD-10: G40, G41
Arterial aneurysm	ICD-10: I71, I72
Uveitis	ICD-10: H20, H30
**Covariates**	
Sex	*The Civil Registration System.*[1] Defined by registered sex.
Age	*The Civil Registration System.*[1] Age was defined by year of study (i.e., year 2024) minus birth year and categorized by 18-39, 40-64, and ≥65 years.
Calendar time	*The Civil Registration System.*[1] Treated as a time-varying covariate in 3-month bins.
Region of residency	*The Civil Registration System.*[1] Defined by the last registered address and categorized according to Northern Denmark Region, Central Denmark Region, Region of Southern Denmark, Capital Region of Denmark, Region Zealand, and unspecified.
Vaccination priority groups	*The Danish Vaccination Register.*[2] The register holds information on all (mandatorily) recorded administered vaccines in Denmark, including information on vaccination date, -type, -dose, and -product batch. In addition, during the Covid-19 pandemic, the register was allocated information on governmentally prioritized Covid-19 vaccine groups assigned according to whether an individual was considered to be at high risk of severe Covid-19 (categorized binarily).
Comorbidities	*The National Patient Register.*[3] The register holds information on all hospital contacts (secondary healthcare facilities) in Denmark including information on the contact duration and treating physician-assigned diagnoses (registered according to the ICD-10-codes). We defined comorbidity status as any registered primary or secondary diagnosis, regardless of the hospital contact type, between 1 January 2019 and baseline (1 May 2024) and indexed by the number of comorbidities as a sum (0, 1, or ≥2 comorbidities).
Asthma	ICD-10: J45, J46
Chronic respiratory disorder	ICD-10: E84, J41-J44, J47, J84
Chronic cardiac disorder	ICD-10: I05-I08, I20-I28, I34-I37, I42-I51
Renal disorder	ICD-10: N03, N05, N07, N18, N19, N25-N27
Diabetes	ICD-10: E10-E14
Autoimmune disorder	ICD-10: D510, D590, D591, D690, D693, D86, E035, E039, E050, E055, E059, E063, E065, E271, E272, E310, G04, G131, G35, G36, G61, G700, H20, I00, I02, K50, K51, K732, K743, K900, L10, L12, L130, L40, L63, L80, M05, M06, M08, M30, M311, M313, M315, M316, M317, M32-M34, M350-M353, M358, M359, M45, M60
Epilepsy	ICD-10 codes: G40, G41
Malignancy	ICD-10 codes: C00-C96 (not C44), D70-D72, D730, D81-D84
Psychiatric disorder	ICD-10 codes: F00-F99

ICD-10 denotes International Classification of Diseases System, version 10, TIA transient cerebral ischemic attack, and NA not applicable. We also excluded individuals with history of the respective outcome under studied before the follow-up period. [a]Only incident outcome events were studied by excluding those individuals with a history of the respective studied outcome during a washout period from 1 January 2019 to study start (1 May 2024) from that specific outcome analysis.

© 2025 Andersson NW et al. *JAMA Network Open*.

eFigure 1. Schematic figure of the study design

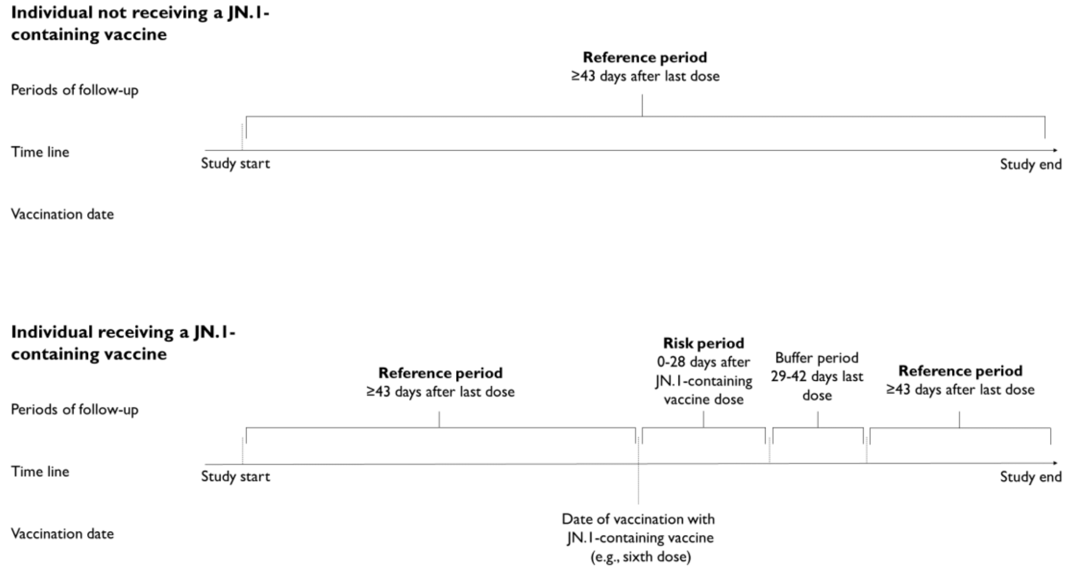

The risk period was from day 0 to day 28 following a vaccination with updated Covid-19 mRNA vaccines containing the SARS-CoV-2 Omicron JN.1 lineage (KP.2 strain). The reference period consisted of a) person-time from the start of the study period (and at least 43 days after any previous Covid-19 vaccine (up until the day before a JN.1-containing vaccine dose) and b) person-time ≥43 days following vaccination with the JN.1-containing vaccine (up until study end). Individuals potentially contributed person-time to both the 28-day risk period and the two reference periods. Day 29-42 period after vaccination was considered a buffer period and thus not included in the reference period. At the end of follow-up (31 March 2025), 1,012,400 of the 1,585,883 included individuals had received a JN.1-containing vaccine (i.e., contributed with both risk and reference period person-time) and 573,483 had not (i.e., contributed with reference period person-time only) during follow-up; the distribution of person-time allocated to the potential risk and reference periods for the two potential follow-ups are presented in the bottom and top panel examples, respectively. During the study period, individuals were followed until the first outcome event (each of the 29 outcomes [see eTable 1] was studied separately) while censoring upon emigration, death, or end of the study period. Outcome rates during the risk and reference periods were compared by incidence rate ratios using adjusted Poisson regression. .

© 2025 Andersson NW et al. *JAMA Network Open*.

eReferences

1. Schmidt, M., Pedersen, L. & Sørensen, H. T. The Danish Civil Registration System as a tool in epidemiology. *Eur J Epidemiol* **29**, 541–549 (2014).
2. Krause, T. G., Jakobsen, S., Haarh, M. & Mølbak, K. The Danish vaccination register. *Euro Surveill* **17**, 20155 (2012).
3. Schmidt, M. *et al.* The Danish National Patient Registry: a review of content, data quality, and research potential. *Clinical Epidemiology* 449 (2015) doi:10.2147/CLEP.S91125.

© 2025 Andersson NW et al. *JAMA Network Open*.
