# Supplementary material for: Safety of JN.1-Updated mRNA COVID-19 Vaccines
Source: JAMA Netw Open. 2025 Jul 28;8(7):e2523557. doi: 10.1001/jamanetworkopen.2025.23557 (PMC12305385; doi:10.1001/jamanetworkopen.2025.23557)
Supplement: Supplement 2. — Data Sharing Statement [file jamanetwopen-e2523557-s002.pdf]

Data Sharing Statement

Andersson. Safety of JN.1-Updated mRNA COVID-19 Vaccines. JAMA Netw Open. Published online July 28, 2025. doi:10.1001/jamanetworkopen.2025.23557

Data
Data available: No

Additional Information
Explanation for why data not available: No additional data available. Owing to data privacy regulations in Denmark, the raw data cannot be shared. However, the data are available for research upon reasonable request to The Danish Health Data Authority and within the framework of the Danish data protection legislation and any required permission from Authorities. Analytical code will be made available on GitHub upon final publication.
